# Sensitivity to sounds in sport-related concussed athletes: a new clinical presentation of hyperacusis

**DOI:** 10.1038/s41598-018-28312-1

**Published:** 2018-07-02

**Authors:** Hussein Assi, R. Davis Moore, Dave Ellemberg, Sylvie Hébert

**Affiliations:** 1 Department of Kinesiology, Université de Montréal, Montreal, Canada; 2 School of Speech Pathology and Audiology, Université de Montréal, Montreal, Canada; 3 International Laboratory for Research on Brain, Music, and Sound (BRAMS), Montreal, Canada

## Abstract

Sensitivity to sounds is one frequent symptom of a sport-related concussion, but its assessment rarely goes beyond a single question. Here we examined sensitivity to sounds using psychoacoustic and psychometric outcomes in athletes beyond the acute phase of injury. Fifty-eight college athletes with normal hearing who either had incurred one or more sport-related concussions (N = 28) or who had never suffered head injury (N = 30) participated. Results indicated that the Concussed group scored higher on the Hyperacusis questionnaire and displayed greater sensitivity to sounds in psychoacoustic tasks compared to the Control group. However, further analyses that separated the Concussed group in subgroups with Sound sensitivity symptom (N = 14) and Without sound sensitivity symptom (N = 14) revealed that athletes with the sound complaint were the ones responsible for the effect: Concussed athletes with self-reported sound sensitivity had lower Loudness Discomfort Thresholds (LDLs), higher Depression and Hyperacusis scores, and shifted loudness growth functions compared to the other subgroup. A simple mediation model disclosed that LDLs exert their influence both directly on Hyperacusis scores as well as indirectly *via* depressive symptoms. We thus report a new clinical presentation of hyperacusis and discuss possible mechanisms by which it could arise from concussion.

## Introduction

Traumatic brain injuries are a significant public health problem, with an estimate of 1.6 to 3.8 million injuries each year in the United States^1^. Sport-related concussions, a specific type of mild traumatic brain injury, are associated with high economic costs^2^. Symptoms of sport-related concussions encompass physical (e.g., headaches, dizziness, troubled vision, and sound sensitivity), emotional (e.g., anxiety, depression), and cognitive (e.g., memory problems) consequences. Although symptoms typically resolve within 7 to 10 days, they persist beyond this acute recovery period in a small but significant proportion of athletes who develop postconcussion syndrome (PCS, i.e., persistence of symptoms between 1 to 3 months after head injury). Predictors of PCS have been proposed, such as prior traumatic brain injury, history of depression, anxiety, attention disorders, multiple injury, as well as sound and light sensitivity^3^. In a study conducted in adults with mild traumatic brain injury (mTBI) mostly from motor vehicle collisions, sensitivity to sounds was the strongest subjective symptom predicting long-term PCS when measured in the acute phase, with a three-fold increased risk at 3 months^4^. Sensitivity to sounds may thus be an indicator of more pervasive neurological dysfunction and of utility for identifying concussed athletes with abnormal recovery profiles. Unfortunately, the evaluation of sensitivity to sounds beyond a single question on the post-concussion symptom scale is largely overlooked in both the clinic and laboratory.

In hearing sciences, sensitivity to sounds pertains to hyperacusis, a general term used to designate a self-reported symptom covering a wide range of reactions to sound such as discomfort or reduced tolerance to sounds: Sounds of moderate intensity are perceived as loud and intrusive^5^. Hyperacusis has no universally accepted definition or manifestation^6^ but is usually assessed using several psychoacoustic and psychometric measures^7,8^. The primary aim of this study was thus to examine the clinical presentation of “sensitivity to sounds” in athletes beyond the acute phase of injury using psychoacoustic (loudness discomfort levels, loudness growth functions) and psychometric (validated hyperacusis questionnaire) outcomes. Second, we aimed to examine how sensitivity to sounds is correlated with other factors such as depressive symptomatology and number of concussions. We predicted that relative to matched teammates without a history of concussion, athletes with a history of concussion would exhibit greater sensitivity to sound. However, since sensitivity to sounds is reported as a symptom in some but not all concussed athletes, we compared concussed athletes with and without sound complaint, in order to better discriminate and characterize these subgroups of concussed athletes.

## Methods

### Participants

Twenty-eight college athletes who incurred one or more sport-related concussions and 30 college athletes who never suffered any type of head injury participated in this study (Table 1). Athletes were recruited from university sports teams (football, rugby, hockey, cheerleading, volleyball), via the university sports-medicine clinic. Exclusion criteria were self-reported history of psychiatric or neurological disease, learning disabilities, attention disorders, alcohol/substance abuse, self-reported inner and/or middle ear pathology (e.g., otosclerosis, cold, otitis media) and pure-tone auditory threshold >15 dB HL at 4 kHz as assessed by the audiometry (see below). All were non-smokers.

**Table 1:** Sociodemographic characteristics of the concussed and control groups.

	Athletes with a history of concussion (N = 28)	Athletes with no history of concussion (N = 30)	*P* value
Male/Female	16/12	14/16	n.s.
Age in years (SD)	21.6 (±2.3)	21.5 (±2.0)	n.s.
Education in years (SD)	15.4 (±2.0)	15.9 (±1.8)	n.s.
Reported tinnitus	4	4	
Number of concussions (SD)	2.04 (±1.0)	—	
Days since last concussion (SD)	38.9 (±17)	—	

In the Concussed group, the three most frequent sports were, in decreasing order, football (n = 11), soccer (n = 7), and rugby (n = 3), while in the Control group sports were rugby (n = 11), football (n = 9), and volleyball (n = 4). Concussions were identified and diagnosed by a physician at the time of injury using the criteria established by the Zurich Guidelines^9^. Specifically, all concussions were identified on the field by the team medical staff, and the attending sports-medicine physician confirmed all diagnoses within 24 hours of injury. At the time of testing concussed athletes were on average 5.6 weeks from injury and had made a complete return to play. All participants were actively participating in their sport at time of testing.

## Materials and Tasks

### Hearing thresholds and loudness discomfort levels (LDLs)

Standard hearing detection thresholds were assessed in each ear monaurally from 0.25 to 8 kHz in octave steps using the standard modified Hughson-Westlake up-down procedure with an AC-40 clinical audiometer and TDH-39 headphones in a soundproof booth. Loudness discomfort levels (LDLs) were assessed monaurally for the same frequencies in increasing 5-dB steps starting at a supra-threshold level. Participants were asked to indicate the level at which the sound was too loud.

### Questionnaires

#### General questionnaire

An in-house questionnaire was used to collect general and medical information as well as information about concussion history and symptoms, such as sensitivity to sound and light, symptom duration, whether symptoms were present or not, etc. Fifty percent (14/28) of concussed athletes reported experiencing sensitivity to sounds following their concussion. Table 2 shows characteristics of the two concussed subgroups according to this complaint.

**Table 2:** Characteristics of the Concussed subgroups reporting sensitivity to sounds and not reporting sensitivity to sounds.

	Reporting sound sensitivity following injury (N = 14)	Not reporting sound sensitivity following injury (N = 14)	*P value*
Male/female	8/6	8/6	n.s.
Sensitivity to light (%)	11 (79%)	3 (21%)	0.007
Symptoms still present (%)	8 (57%)	0 (0%)	0.002
Symptom duration in days (SD)	32.5 (13)	11.2 (12)	0.001
Number of concussions (SD)	2 (0.9)	2 (1.0)	n.s.
Days since last injury (SD)	40 (11)	37 (22)	n.s.
Reported tinnitus (%)	4 (29%)	0 (0%)	0.031

#### Hyperacusis

This questionnaire has 14 items assessing auditory sensitivity to external sounds. Each item is rated on a 4-point scale, ranging from “no” (0 points) to “yes, a lot” (3 points) and yields a total score between 0 and 42^10^.

#### Depressive symptomatology

The Beck Depression Inventory-II (BDI-II) is a 21-item^11^ questionnaire assessing the severity of depressive mood in participants over the last two weeks, including today. Each item is rated from 0 (Not at all) to 3 (Severely), yielding a possible total score between 0 and 63.

#### Anxiety

The Beck Anxiety Inventory (BAI) is a 21-item self-report assessing the severity of anxiety over the last week, including today. Each item is rated on a scale of 0 (Not at all) to 3 (Severely) and can yield a total score between 0 and 63^12^.

#### Loudness growth functions

**Figure 1:**
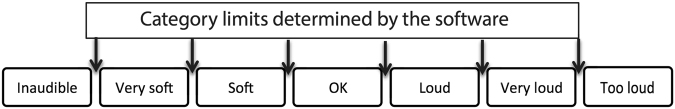
The seven loudness categories used to judge the stimuli in the psychophysical loudness function task. Arrows indicate the six category limits determined by the software.

Loudness growth functions were assessed in each participant using an adaptive psychophysical loudness function task. The task determines the six boundaries lying between seven loudness categories from Inaudible to Too Loud and identified as *Very Soft, Soft, Ok, Loud, Very Loud, and Too Loud* (Fig. 1), thus representing the lower limit of each category. Trains of three frequency-modulated 4-kHz tones were presented binaurally using DT 770 PRO headphones (Beyerdynamics Ltd) at different levels and participants had to judge each trial using the above loudness categories. The sound level was automatically increased or decreased depending on the participants’ response and varied in steps from 5 dB to 1 dB, bracketing the boundary of each tested category. The task ends when five 1-dB steps reversals have been completed for all boundaries. In order to get valid and comparable measures, a common definition for “Too Loud” was presented to participants as corresponding to a sound above the level to which a person would not choose to listen for any period of time.

### Procedure

Participants were tested individually. Upon entering the laboratory, participants completed a medical and concussion history questionnaire, followed by the Hyperacusis, BDI-II and BAI questionnaires. Participants then completed the hearing tests. Psychophysical testing was performed using Sennheiser HD265 headphones calibrated with a SoundPro SE/DL sound level meter using a QE-4170 microphone model (Quest Technologies, Oconomowoc, WI, USA) and an EC-9A 2cc ear coupler (Quest Electronics, Oconomowoc, WI, USA). Testing took about 50 minutes. The study was conducted with the approval of the ethics committee of Université de Montréal and all participants gave their informed written consent. The experiment was performed in accordance with relevant guidelines and regulations.

### Data analyses

Preliminary analyses with sex as a between-subject factor did not reveal any relevant main effects or interactions on loudness functions and questionnaires, and thus data were collapsed across this factor and was not further considered. In all analyses below, the two groups (Concussed vs. Controls) were first compared, and in a second step the Concussed group was subdivided based on their self-reported sound sensitivity into Concussed with sound sensitivity symptom (Sound sensitive, or SS) and Concussed without sound sensitivity symptom (Without sound sensitivity subgroup, or WSS). Hearing thresholds (HT) and loudness discomfort levels (LDL) were analyzed separately with mixed ANOVAs with Group (1^st^ Concussed vs. Controls; 2^nd^ SS vs. WSS) as a between-subject factors and Ear (Right, Left) and Frequency (250 Hz – 8 kHz) as within-subject factors. Mean questionnaire scores were analysed using *t*-tests. Loudness functions were analysed using mixed ANOVAs with Group (1^st^ Concussed vs. Controls; 2^nd^ SS vs. WSS) as a between-subject factors and Category limits as within-subject factors. Pearson product-to-moment correlations between LDL at 4 kHz (i.e., the same frequency as in Loudness growth functions), dB level for the Too loud level category, scores on the Hyperacusis, BDI-II and BAI questionnaires, and number of concussions, were conducted on all data. Following correlations, a mediation model was tested to predict how LDLs exert their influence on the subjective complaint (scores on the Hyperacusis questionnaire), with depressive symptoms (BDI-II scores) as a mediator. To this aim, we calculated the direct, indirect, and total effects of predictors using PROCESS v3.0^13^. We used bootstrapping analyses of the sampling distribution (with N = 5,000 bootstrap re-samples) to test the indirect effects as suggested by Hayes^13^. A 95% confidence interval was calculated around the parameter estimate, which was considered significant when the 95% CI did not cross zero.

## Results

### Hearing thresholds and Loudness Discomfort Levels (LDLs)

**Figure 2:**
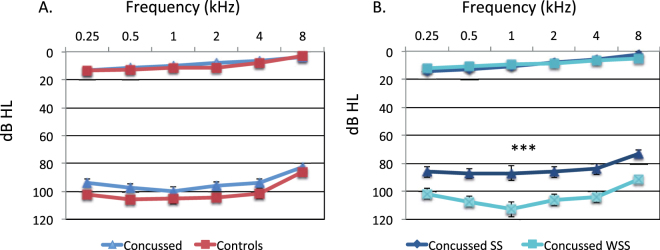
Hearing thresholds and LDLs in dB HL for frequencies 250 to 8 kHz for the Concussed and Control groups (panel A, triangle and square symbols, respectively) and for the Concussed with (SS) or without (WSS) self-reported sound sensitivity (panel B, diamond and square symbols, respectively). LDLs differed between the two concussed subgroups. ***p = 0.001.

There was no difference in hearing thresholds between groups, *F* (1,56) = 1.49, *p* = 0.23, nor was any interaction of group with ear or frequency, both *p*s > 0.70 (Fig. 2 panel A). A similar pattern was found when the two subgroups were compared, *F* < 1 (Fig. 2 panel B). The Concussed group had lower LDLs than the Controls, but the main effect of Group did not reach significance, *F* (1, 56) = 3.58, *p* = 0.07, means of 93.6 (2.8) and 100.9 (2.7), respectively. When considering the Concussed subgroups, however, the Sound sensitive subgroup had significantly lower LDLs than those Without sound sensitivity (mean SS = 84, mean WSS = 103 dB HL, *F* (1, 26) = 15.21, *p* = 0.001).

### Questionnaires

**Figure 3:**
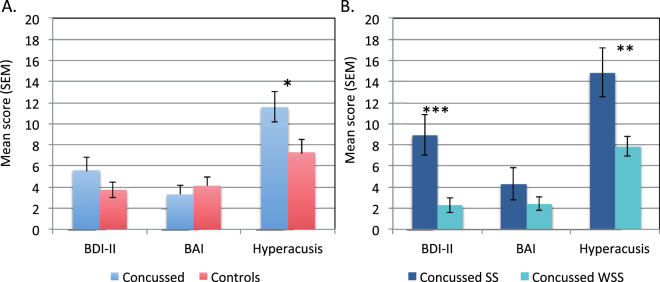
Mean scores on Questionnaires (SEM) for the Concussed and Control groups (panel A) and for the Concussed with (SS) or without (WSS) self-reported sound sensitivity (panel B). *p = 0.03 **p = 0.01 ***p = 0.003.

Although the Concussed group reported higher scores on all questionnaires (Fig. 3), group differences only reached significance only for the Hyperacusis questionnaire (*m*NS = 11.4, *m*WNS = 7.3; *t* (55) = 2.23, *p* = 0.03). Mean BDI-II scores were 5.6 and 3.8 for the two groups, respectively (*p* = 0.19), and means for the BAI were 3.3 and 4.1, respectively (*p* = 0.49).

When considering the Concussed subgroups, the Sound sensitive subgroup had significantly higher reported depressive symptoms BDI-II (*m*SS = 8.9, *m*WSS = 2.3, *p* = 0.003) and Hyperacusis scores (*m*SS = 14.9, *m*WSS = 7.9; *p* = 0.01) compared to the other one. Subgroups did not differ significantly on the BAI questionnaire (means = 4.3 vs. 2.4, *p* = 0.25).

### Loudness growth functions

**Figure 4:**
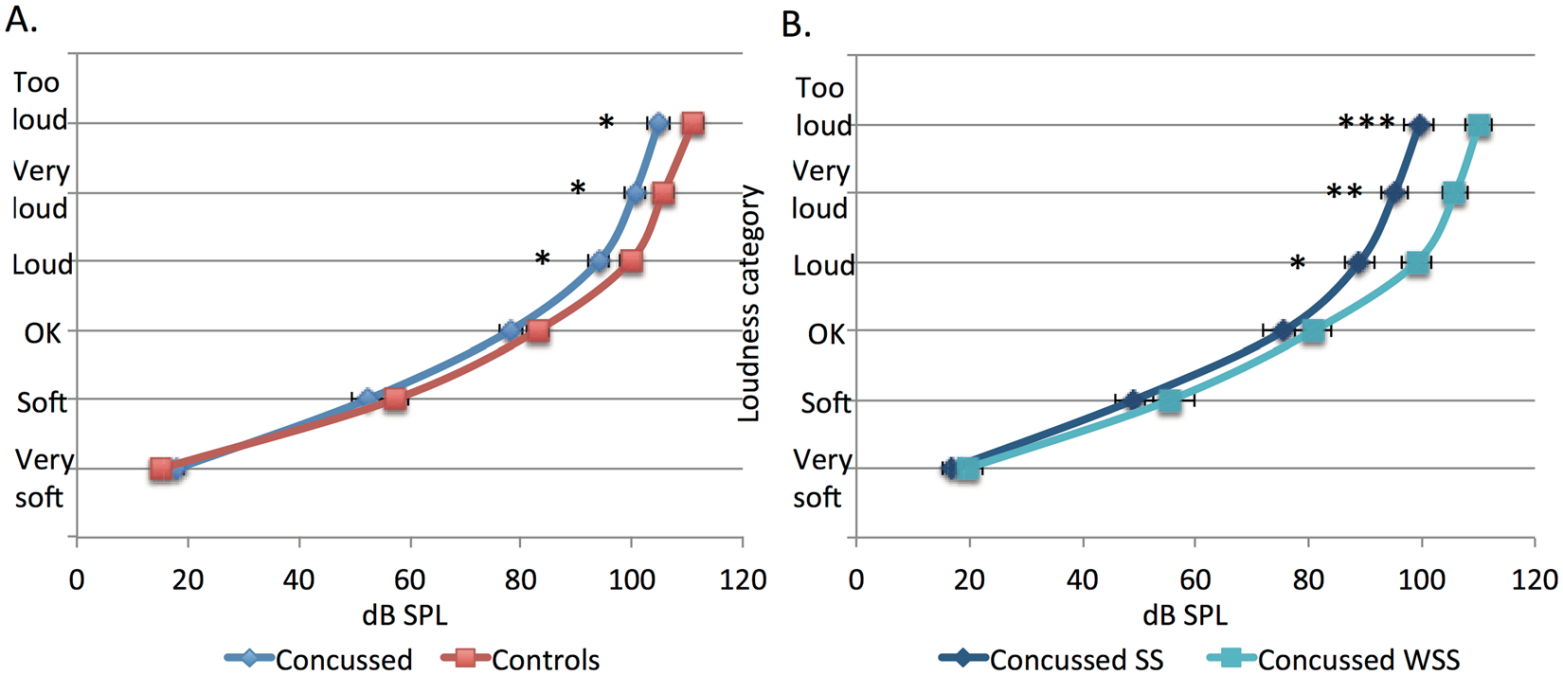
Loudness growth functions (SEM = Standard Error of the Mean) for the Concussed and Control groups (panel A) and for the Concussed with (SS) or without (WSS) self-reported sound sensitivity (panel B). *p < 0.05 **p = 0.004 ***p = 0.005.

The expected interaction between Group and Category was significant, *F*(5,280) = 2.68, *p* = 0.02 (Fig. 4). Loudness levels were all lower in the Concussed compared to Controls in all categories. However, independent t-tests revealed that these differences were significant only for the Loud (94 vs 99 dB, *p* = 0.03), Very Loud (101 vs. 106 dB, *p* < 0.05), and Too Loud categories (105 vs. 111 dB, *p* < 0.03). No significant effects were observed for the Very Soft (18 vs. 15 dB, *p* = 0.12), Soft (52 vs. 57 dB, *p* = 0.19), and Ok categories (78 vs. 83 dB, *p* = 0.10).

When considering the two Concussed subgroups, the Sound sensitive subgroup showed differences of ~10 dB from the other subgroup for the Loud (89 vs. 99 dB, *p* = 0.013), Very Loud (95 vs. 105 dB, *p* = 0.004), and Too Loud categories (100 vs. 110 dB, *p* = 0.005), but did not differ for the categories Very Soft (17 vs. 19 dB, *p* = 0.39), Soft (49 vs. 55 dB, *p* = 0.26), and Ok (76 vs. 81 dB, *p* = 0.29), by independent t-tests. Removing the data from the four athletes with tinnitus in the Sound sensitive subgroup from this analysis did not change the results.

The loudness growth functions of Concussed subgroup Without sound complaint did not differ from those of the Controls (all *p*s from 0.09 to 0.96 by independent t-tests).

### Correlations (all data)

Loudness Discomfort Levels at 4 kHz were strongly correlated with Too loud dB levels (see Table 3), and moderately negatively correlated with BDI-II and Hyperacusis scores (all *ps* ≤0.001), meaning that the higher the depressive symptoms and Hyperacusis scores, the lower the LDL levels. LDLs were not correlated with BAI scores (*p* = 0.11). Too Loud levels showed similar correlations, with the additional nearly significant correlation with number of concussions (*p* = 0.06). Hyperacusis and BDI-II scores were moderately correlated with one another, *r* (54) = 0.541, *p* < 0.001, and the same was true for Hyperacusis and BAI scores, *r* (54) = 0.564, *p* < 0.001, with higher Hyperacusis scores associated with higher depressive symptomatology and anxiety. The correlation between the number of concussions and Hyperacusis just failed to reach significance (*p* = 0.058), thus showing a trend towards more concussions being associated with lower dB levels in the Too Loud levels and higher hyperacusis scores. All significant correlations survived the Bonferroni correction (0.05/15 = 0.003) for multiple correlations.

**Table 3:** Pearson product-to-moment correlation values between LDL at 4 kHz, dB level for the Too loud category, scores on questionnaires, and number of concussions.

	LDL at 4 kHz	Too loud Level (dB)	Hyperacusis scores	BDI-II scores	BAI scores	Number of concussions
LDL at 4 kHz	—	0.797***	−0.518***	−0.444***	−0.213	−0.221
Too Loud Level		—	−0.556***	−0.400**	−0.184	−0.248
Hyperacusis scores			—	0.541***	0.564***	0.253
BDI-II scores				—	0.634***	0.074
BAI Scores					—	−0.097

***p ≤ 0.001; **p ≤ 0.002.

### A simple mediation model

**Figure 5:**
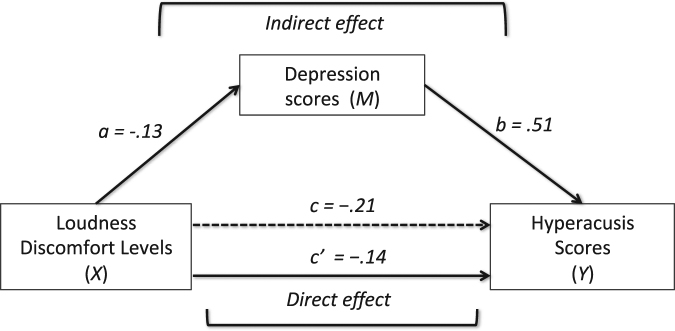
Mediation model and unstandardized parameters estimate for each path. LDLs exert an effect on Hyperacusis scores both directly (c’) and indirectly through Depression scores (ab). The total effect (c) is the sum of the direct (c’) and indirect (ab) effects.

Previous studies have shown poor or inconsistent correlations between LDLs and Hyperacusis scores^14–16^, possibly because hyperacusis questionnaires assess tolerance of sounds experienced in everyday life that have little physical resemblance with the 4 kHz pure tone used in the LDL assessment. In addition, since LDLs are associated with both self-reported hyperacusis and depressive symptoms^17–20^, as found here, and depression is correlated with hyperacusis, it is possible that LDLs can be explained by depressive symptoms, which results in a complaint of hyperacusis. Regression analysis was thus used to investigate the hypothesis that depressive symptoms mediate the effect of LDLs on Hyperacusis scores. To this aim we tested a simple mediation model (Fig. 5) in which LDLs (X) exert their influence directly (path c’) on Hyperacusis scores (Y) or indirectly (path a* path b) through depressive symptoms (M).

Results showed that the total effect of LDLs on Hyperacusis scores (path c, i.e., regression of LDLs on Hyperacusis scores, ignoring depressive symptoms), was significant (b = −0.21, *F*(1,54) = 21.29, *p* < 0.0001). The effect of LDLs on depressive symptoms (path a) was also significant (b = −0.13, *F*(1, 54) = 13.50, *p* = 0.0005), and so was the effect of depression on Hyperacusis scores, while controlling for LDLs ((path b), b = 0.51, *t*(54) = 3,18, *p* = 0.0024). Approximately 40% of the variance in the Hyperacusis questionnaire was accounted for by the indirect effect (R^2^ = 0.398). The indirect effect (ab) tested using a bootstrap estimation approach with 5,000 samples was significant, b = −0.0666, SE = 0.0340, 95% CI = −0.1360, −0.0064.

In addition, when controlling for depression (direct effect, or path c’), LDLs was still a significant predictor of Hyperacusis scores (b = −0.14, *t*(54) = −3,04, *p* = 0.0037). In summary, LDLs exert their influence on hyperacusis both directly and indirectly through depressive symptoms.

## Discussion

We report a new clinical presentation of hyperacusis (self-reported symptom of sensitivity to sounds) that is related to a specific form of mild traumatic brain injury, namely sport-related concussions, and that is not related to tinnitus^21–24^, acoustic shock or trauma^25,26^, stroke^27,28^, focal cortical damage^29,30^, loudness recruitment caused by hearing loss^31^, Williams Syndrome^32,33^, Lyme disease^34^, or multiple sclerosis^35^. Although hyperacusis has been reported in some traumatic brain injury patients^3,36^, diverse etiologies (e.g., motor vehicle accident, blow to the head) and other potentially confounding auditory symptoms (e.g., hearing loss, tinnitus) prevent assumptions about its origin and pathophysiology. In contrast, the homogeneous etiology of hyperacusis reported herein with normal peripheral hearing and absence −or irrelevance− of tinnitus suggests that the reported sound sensitivity is directly related to the mechanisms of sport-related concussion.

To summarize our findings, concussed athletes beyond the phase of acute injury with normal detection thresholds and a complaint of sensitivity to sounds exhibited loudness discomfort levels for standard audiometric frequencies (250 Hz to 8 kHz) as well as reduced sound tolerance of ~10 dB for Loud, Very Loud, and Too Loud sound categories compared to concussed athletes not reporting sound sensitivity and athletes without concussion. Since a 10 dB difference in level represents a change in power by a factor of 10, it is striking that a sound judged as barely Loud in the concussed athletes without sound complaint and in controls (99 dB, representing the limits between OK and Loud) was judged as Too Loud by those with sound sensitivity (100 dB), that is, two loudness categories above. This decreased sound tolerance was associated with higher depressive symptomatology and Hyperacusis scores, and marginally with number of concussions. Subgroups differed in the mean duration of their symptoms in a ~3:1 ratio (32.5 days vs. 11.2 days for the subgroups with and without sound complaint, respectively). Consistent with some previous data, sound sensitivity may be indicative of more pervasive and complex neurological sequalae following concussion. In addition, mediation analyses showed that LDLs, as measured with pure tones in an audiometric booth, exert both a direct influence on the complaint of sound sensitivity in everyday life (e.g., Hyperacusis questionnaire) and an indirect one via depressive symptoms. This suggests two different possible pathways by which sound sensitivity complaints can arise in concussed athletes, namely, via the physical and the psychological consequences of concussions.

Sound intensity is first coded at the periphery of the auditory system by the basilar membrane. Increased sound pressure increases the amplitude of the basilar membrane displacement, which in turn increases the spike rates in primary afferent auditory nerve fibers and the number of responding fibers^37^. High intensity sounds are coded by high-thresholds fibers and produce greater vibration and greater rate of action potentials going to the brain. The total neural activity determines loudness, which is coded in the auditory cortices^38^. Currently, hyperacusis is best explained by the *central gain model*, in which peripheral damage, i.e., hearing loss, is (over)corrected by increased spontaneous and sound-evoked firing rates: increased spontaneous firing rates would give rise to tinnitus, which classically mirrors hearing loss^8,39–41^, while increased sound-evoked firing rates would give rise to hyperacusis. Increased auditory cortical spontaneous and evoked firing rates have been reported in animal models of tinnitus using noise-induced hearing loss^42^. Yet this *homeostatic* plasticity involves a common mechanism between tinnitus and hyperacusis that stem from peripheral damage^21^ and thus cannot account for the clinical presentation of hyperacusis here. In order to involve homeostatic mechanisms such as central gain, one has to postulate that cortical auditory spontaneous and sound-evoked firing rates can work independently (as for instance when salicylate is applied systemically^43^) and directly at the central level, i.e., without peripheral damage. We therefore suggest that the type of hyperacusis found here is directly related to central biochemical, mechanical, and inflammatory brain responses subsequent to concussion.

Concussion entails linear and rotational acceleration of the brain and its ensuing biochemical and neuronal disruptions. The chain of reaction includes spreading depolarization, a term for the whole spectrum of waves in the central nervous system characterized by abrupt, near-complete sustained depolarization of neurons, observed as a large change of the slow potential^44^. Neurotransmitter dysregulation, neuroinflammatory responses, cerebral blood flow changes, diffuse axonal injury, all of these can all lead to chronic pathophysiology^45^. In particular, hyperacute release of the excitatory neurotransmitter glutamate and changes in the inhibitor neurotransmitter g-aminobutyric acid (GABA) and its receptors^45,46^, combined with a persistent and sustained neuroinflammatory response, can significantly disrupt the excitatory-inhibitory neural balance and produce increased neural responses to sounds in the auditory cortices. Interestingly, sensitivity to sounds was accompanied by self-reported sensitivity to light in 80% of athletes. Since perceived brightness is represented in the responses of neurons in striate (visual) cortices as part of a neural representation of object surfaces^47^, and that more perceived brightness is associated with more neural activity, sensitivity to sound and light might share some common mechanisms related to increased neural activity and /or excitatory-inhibitory imbalance in the primary sensory cortices when stimuli reach a critical amount of recruited fibers. In any case, given the presentation of hyperacusis reported here, new models of hyperacusis unrelated to tinnitus should be proposed and developed in the near future.

In conclusion, sound sensitivity starting at dB levels that most people find barely loud can represent an important disabling symptom in concussed athletes, especially when some depressive symptoms are present. Because symptoms and loss of activity are reported as the worst part of concussion by athletes^48^, it is important to realize that further social isolation and sensory deprivation can lead to increased symptoms^49^. Although there is no universally accepted treatment for hyperacusis, one therapy that has received some empirical support is cognitive behavioural therapy^7^, which is also successful for treating patients with subclinical depressive symptoms such as athletes in the present study^50^. Given the new etiology of hyperacusis presented here and the fact that it is associated with pervasive abnormal and/or prolonged recovery, and given the relative ease of administering and availability of sensory sensitivity questionnaires, clinicians and health professionals should be encouraged to identify and assess this prevalent symptom in the audiology clinic and guide athletes to appropriate follow-up care.
